# Paradoxical DNA Repair and Peroxide Resistance Gene Conservation in *Bacillus pumilus* SAFR-032

**DOI:** 10.1371/journal.pone.0000928

**Published:** 2007-09-26

**Authors:** Jason Gioia, Shailaja Yerrapragada, Xiang Qin, Huaiyang Jiang, Okezie C. Igboeli, Donna Muzny, Shannon Dugan-Rocha, Yan Ding, Alicia Hawes, Wen Liu, Lesette Perez, Christie Kovar, Huyen Dinh, Sandra Lee, Lynne Nazareth, Peter Blyth, Michael Holder, Christian Buhay, Madhan R. Tirumalai, Yamei Liu, Indrani Dasgupta, Lina Bokhetache, Masaya Fujita, Fathi Karouia, Prahathees Eswara Moorthy, Johnathan Siefert, Akif Uzman, Prince Buzumbo, Avani Verma, Hiba Zwiya, Brian D. McWilliams, Adeola Olowu, Kenneth D. Clinkenbeard, David Newcombe, Lisa Golebiewski, Joseph F. Petrosino, Wayne L. Nicholson, George E. Fox, Kasthuri Venkateswaran, Sarah K. Highlander, George M. Weinstock

**Affiliations:** 1 Human Genome Sequencing Center, Baylor College of Medicine, Houston, Texas, United States of America; 2 Department of Molecular and Human Genetics, Baylor College of Medicine, Houston, Texas, United States of America; 3 Department of Molecular Virology and Microbiology, Baylor College of Medicine, Houston, Texas, United States of America; 4 Department of Biology and Biochemistry, University of Houston, Houston, Texas, United States of America; 5 Department of Natural Sciences, University of Houston‐Downtown, Houston, Texas, United States of America; 6 University of St. Thomas, Houston Texas, United States of America; 7 Department of Veterinary Pathobiology, Center for Veterinary Health Sciences, Oklahoma State University, Stillwater, Oklahoma, United States of America; 8 University of Idaho Coeur d'Alene, Coeur d'Alene, Idaho, United States of America; 9 Jet Propulsion Laboratory, California Institute of Technology, Pasadena, California, United States of America; 10 Department of Microbiology and Cell Science, University of Florida Space Life Sciences Laboratory, Kennedy Space Center, Florida, United States of America; Tufts University, United States of America

## Abstract

**Background:**

*Bacillus* spores are notoriously resistant to unfavorable conditions such as UV radiation, γ-radiation, H_2_O_2_, desiccation, chemical disinfection, or starvation. *Bacillus pumilus* SAFR-032 survives standard decontamination procedures of the Jet Propulsion Lab spacecraft assembly facility, and both spores and vegetative cells of this strain exhibit elevated resistance to UV radiation and H_2_O_2_ compared to other *Bacillus* species.

**Principal Findings:**

The genome of *B. pumilus* SAFR-032 was sequenced and annotated. Lists of genes relevant to DNA repair and the oxidative stress response were generated and compared to *B. subtilis* and *B. licheniformis*. Differences in conservation of genes, gene order, and protein sequences are highlighted because they potentially explain the extreme resistance phenotype of *B. pumilus*. The *B. pumilus* genome includes genes not found in *B. subtilis* or *B. licheniformis* and conserved genes with sequence divergence, but paradoxically lacks several genes that function in UV or H_2_O_2_ resistance in other *Bacillus* species.

**Significance:**

This study identifies several candidate genes for further research into UV and H_2_O_2_ resistance. These findings will help explain the resistance of *B. pumilus* and are applicable to understanding sterilization survival strategies of microbes.

## Introduction

*Bacillus pumilus* is a Gram-positive, aerobic, rod-shaped, soil-dwelling bacterium [1]. Like other *Bacillus* species, *B. pumilus* produces spores that are more resistant than vegetative cells to heat, desiccation, UV radiation, γ-radiation, H_2_O_2_, and starvation. *B. pumilus* has been found in extreme environments such as the interior of Sonoran desert basalt and the Mars Odyssey spacecraft [2], [3]. Spores and vegetative cells of *B. pumilus* SAFR-032, a strain originally recovered from the Jet Propulsion Lab (Pasadena, CA) spacecraft assembly facility, are endowed with UV radiation and H_2_O_2_ resistance capabilities that significantly exceed other *Bacillus* species and allow survival of standard sterilization practices [3]–[5]. Sterilization is significant not only for prevention of contamination of extraterrestrial environments via spacecraft, but also for fundamental processes in bacteriology, medicine, the pharmaceutical industry, and counter-bioterrorism measures, and hence such resistance is cause for concern.

UV radiation induces the formation of deleterious DNA lesions such as pyrimidine dimers [5], [6]. *Bacillus* spores are more resistant to UV radiation than vegetative cells because desiccation and the presence of small acid soluble spore proteins (SASP) mitigate DNA damage. A variety of DNA repair mechanisms that become active upon germination also permit survival of UV radiation. H_2_O_2_ kills spores by oxidative damage to the inner membrane and it also causes oxidative damage to cellular proteins and DNA [7], [8]. H_2_O_2_-induced damage is combated by a variety of reducing agents that react with oxidative agents or oxidized cellular components.

Here we present an analysis of the *B. pumilus* SAFR-032 genome. In comparing this genome to less UV- and H_2_O_2_-resistant *Bacillus* species (*B. subtilis* and *B. licheniformis*) we identify genomic differences that provide important insights into the DNA repair pathways and oxidative stress response pathways of *B. pumilus*. The genes identified in this study are candidates for further experimental research.

## Methods

### Bacterial strain growth and DNA isolation

A single *B. pumilus* SAFR-032 colony exhibiting circular, crateriform morphology and raised ridges on its surface, was used to inoculate trypticase soy yeast (TSY) broth. The culture was grown overnight at 37°C with vigorous shaking. Genomic DNA was purified from CsCl gradients of whole cell lysates [9].

### DNA sequencing and genome assembly

DNA sequencing was performed by a combined approach using traditional Sanger dideoxy whole genome shotgun (WGS) sequencing and 454 Life Sciences pyrosequencing strategies [10]. Genomic DNA was nebulized into 5 kb fragments, and cloned into a derivative of pUC18 [11]. The clones were used for WGS DNA sequencing using ABI 3700 sequencers, and reads were assembled using the ATLAS assembler [12]. Read-pair information was used to create higher order scaffolds. WGS reads were sequenced to ten-fold coverage. The WGS plasmid libraries were not random, but had cloning bias of unknown cause. Consequently, the WGS sequence was supplemented with short reads generated on a 454 Life Sciences GS20 sequencer and lacking cloning bias. Here the coverage was thirteen fold.

### Gene identification and annotation

Previously described gene prediction and manual annotation protocols were followed [13]. Glimmer [14] and GeneMark [15] were used independently to predict open reading frames (ORFs). Visualization of gene predictions was performed using the Genboree system (www.genboree.org) and the CONAN database [13]. DNA comparisons were performed with BLASTN and BLASTZ. Protein sequences were analyzed by BLASTP vs. the nr database at NCBI [16]. When appropriate, other predictive tools such as InterProScan [17], PFP [18], PSORTb [19], ExPASy ENZYME [20], Helix-Turn-Helix Predictor [21], MEROPs [22], and the Transport Classification Database [23] were used. The *B. pumilus* SAFR-032 genome is 3.7 Mb and 3848 features (3687 ORFs, 12 frameshifts, 38 pseudogenes, 7 rRNA operons, 69 tRNAs, and 21 ncRNAs) were annotated. The *B. pumilus* genome has been deposited in GenBank under the accession number CP000813. Locus tags of genes discussed in this paper are listed in Supplementary Table S1.

### Comparative Genomic Analysis

The database of annotated genes was searched for genes relevant to DNA repair and H_2_O_2_ resistance. *B. pumilus* genes were considered homologs of *B. subtilis* and *B. licheniformis* genes if their translated sequences aligned with ≥50% identity to the homolog of either species. Exceptions were made in deference to conserved gene order and local alignments to functional domains characteristic of specific proteins. We examined the *B. subtilis* and *B. licheniformis* genomes and available literature to find DNA repair and H_2_O_2_ resistance genes not found in our *B. pumilus* gene list. Relevant genes absent from the *B. pumilus* gene list were confirmed as absent using *B. subtilis* and *B. licheniformis* sequences as queries for local BLAST against the *B. pumilus* genome.

### Spore survivability to UV radiation and H_2_O_2_

Methods of measuring survival of spores exposed to UV radiation and H_2_O_2_ have been previously described [3], [4]. Data presented here include but are not limited to measurements previously reported in those studies.

## Results and Discussion

**Figure 1:**
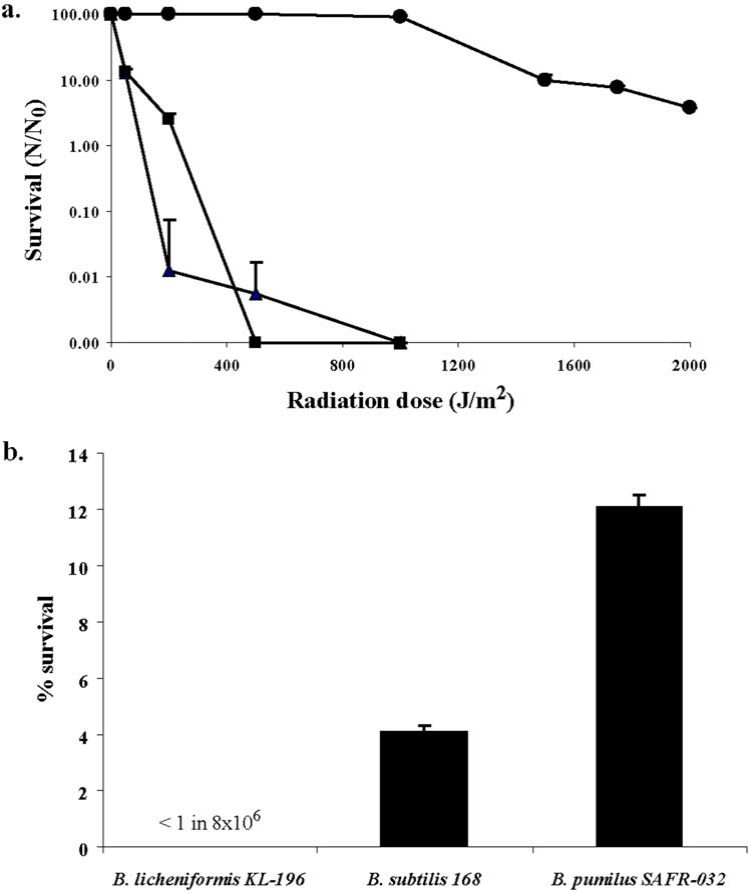
Resistance of *B. pumilus* SAFR-032 spores to UV radiation and H_2_O_2_. a) Survivability of spores exposed to varying doses of UV254 (100 uW sec^−1^cm^−2^). Key: *B. pumilus* SAFR-032, circles; *B. subtilis* 168, squares; *B. licheniformis* ME-13-1, triangles. b) Survivability of spores exposed to 5% H_2_O_2_ liquid for one hour.

*B. pumilus* SAFR-032 was selected for genome sequencing and analysis because its spores exhibited unusually high resistance to UV radiation and H_2_O_2_ compared to the standard dosimetric strains *B. subtilis* 168 and *B. licheniformis*. Whereas >90% lethality of *B. subtilis* and *B. lichenifiormis* spores is achieved by exposure to 200 J/m^2^ UV254, 1500 J/m^2^ are required to kill 90% of *B. pumilus* SAFR-032 spores (Figure 1a). Twelve percent of *B. pumilus* SAFR-032 spores survive 5% liquid H_2_O_2_, which is nearly thrice the survival rate of *B. subtilis* spores (Figure 1b).

The *B. pumilus* SAFR-032 genome was annotated and analyzed for features relevant to UV radiation resistance and H_2_O_2_ resistance. Mechanisms of DNA repair and the oxidative stress response were compared among *B. pumilus*, *B. subtilis*, and *B. licheniformis* to generate lists of genes common to all three species, genes unique to *B. pumilus*, and genes absent in *B. pumilus* (Table 1). The presence or absence of genes is indicative of unique functions that may explain phenotypic differences. Despite gene conservation, the possibility of altered functions of homologous genes due to sequence divergence cannot be excluded. Therefore, the translated sequences of common genes were also compared (Tables 2 and 3). In addition to gene conservation and sequence similarity it is also important to understand gene functions in context of the organism's growth phase. Although the temporal activity of only some proteins discussed here are known, two recent studies describe transcription of many *B. subtilis* DNA repair and H_2_O_2_ resistance genes. Keijser et al. identified transcripts more abundant in spores and germinating cells than in vegetative cells [24]. Moeller et al. identified transcripts induced after exposure of vegetative cells to UVC radiation (200–280 nm) [25]. We cross-referenced our gene lists with these temporal transcription data to augment our genomic comparisons (Table 1). However, it should be understood that because spore survivability assays entail growing surviving spores to countable levels in liquid or solid media, resistance mechanisms at any stage of growth may be important to survivability.

**Table 1:** List of *Bacillus* genes involved in DNA repair and oxidative stress resistance.

Function (No. of genes)	Class	Gene	Missing in Bp [1]	Missing in Bl [2]	Missing in Bs [3]	Missing in Bp & Bl [1], [2]	Missing in Bl & Bs [2], [3]
DNA repair (88)	U	*ada* ^2,3^; *dinG*; *disA*; *dnaE*; *end1*; *gyrB*; *hbs*; *kapD*; *mfd*; *mutL*,*M*,*S2*(*yshD*); *ogt*; *pcrB*; *phrB* ^2,3^; *polA*; *polY2(yqjW)*; *priA*; *recD*, *J*, *N*,*R*,*Q*(*recS*),*X*; *sbcC*,*D*; *scpA*,*B*; *sms*; *uvrX^1^*; *xseA*,*B*; *yjhB^1^*;*ykoW*; *ylbH*; *yocI*; *yobH* ^1,2^; *yorK* ^1,2^; *yozK* ^1,2^; *yqfN*; *yrrK*; *yvcI; ywbD*	5	5	2	3	2
	V	*alkA*; *dinB* ^1^; *lexA*; *mutS1*; *polY1(yqjH); radC; recF,G,O; ruvA*,*B; ssb; yjcD*; *yneB*; *ywjD* ^2^(*uvsE*)	1	1	0	0	0
	G&O	*addA,B; exoA* ^1,2^; *gyrA; mutT* ^1^,*Y*; *nth; pcrA; recU; topA*; *ung*; *uvrC*; *ydiP* ^2^; *yhaZ* ^2^ *; ypcP*; *yprA*; *ypvA*; *yrrT; yrvN*; *ywqA*, *yxlJ* ^1^	3	3	0	1	0
	S	*nfo*; *yqhH*	0	0	0	0	0
	V+G&O	*recA; uvrA*,*B; ykoU*(*lig*),*V*(*ku*)	0	0	0	0	0
	S+G&O	*splA*,*B*	0	0	0	0	0
Oxidative stress resistance (35)	U	*bcrC; cotJC* ^1^; *katX2* ^2,3^; *msrA*; *ohrA*,*B*,*R*; *sigM*; *sodF*; *trxA*; *ycgT*; *ygaF*; *yjqC*; *ykuU*; *ylaC* ^1^,*D* ^1^;*yojM*; *yqjL*	3	1	1	0	1
	V	*mrgA* ^1^; *msrB*	1	0	0	0	0
	G&O	*ahpC* ^1^,*F* ^1^; *bsaA*; *perR*; *sigB*; *sodA*; *spx*; *tpx*; *trxB; ydbD* ^1^; *yqjM*	3	0	0	0	0
	V+G&O	*dpsA*; *katA* ^1^,*B* ^1^(*katE*)	2	0	0	0	0
	S+G&O	*katX(yxlI)*	0	0	0	0	0
SASP (18)	S	*csgA* ^1^; *sspA*,*B*,*C* ^1,2^,*D*,*E*,*F*,*G* ^1,2^,*H* ^1^,*I*,*J*,*K*,*L*,*M*,*N*,*O* (*cotK*),*P*(*cotL*);*tlp*	4	2	0	2	0

1 = absent in *B. pumilus* SAFR-032 (Bp). 2 = absent in *B. licheniformis* (Bl). 3 = absent in *B. subtilis* 168 (Bs).

U = expression unknown.

V = genes transcribed in *B. subtilis* vegetative cells [25].

G&O = genes transcribed during *B. subtilis* spore germination & outgrowth [24], [42], [70].

S = gene products present in *B. subtilis* spores [26], [34], [42].

**Table 2:** Sequence conservation of DNA repair proteins among *Bacillus* species.

Pathway	Protein	% identity Bp vs. Bs	% identity Bp vs. Bl	% identity Bs vs. Bl
Base excision repair				
	AlkA (YfiP)	60	68	78
	Ung	73	73	83
	MutM (Fpg)	64	63	77
	MutY (YfhQ)	68	66	75
	Nth	88	88	89
	Nfo (YqfS)	86	87	91
Nucleotide Excision Repair				
	Mfd	80	81	85
	PcrA	83	85	87
	UvrA	86	87	86
	UvrB	89	89	92
	UvrC	82	84	84
Mismatch Repair				
	MutS	79	78	82
	MutL	77	73	78
	XseA (YqiB)	73	71	75
	XseB (YqiC)	74	77	73
NHEJ				
	YkoU	40	41	58
	YkoV	49	52	68
	YkoW	49	34	30
Homologous recombination				
	AddA	66	67	72
	AddB	64	65	74
	LexA	87	87	91
	PriA	71	72	79
	RecA	93	95	93
	RecD (YrrC)	79	82	79
	RecF	84	84	89
	RecG (YlpB)	81	80	81
	RecJ (YrvE)	67	62	68
	RecN	74	75	81
	RecO	75	77	78
	RecQ (RecS)	53	53	58
	RecR	97	98	98
	RecU	70	73	80
	RecX (YfhG)	63	64	70
	RuvA	81	77	84
	RuvB	85	85	88
	SbcC (YirY)	51	49	56
	SbcD	74	74	78
Spore Photoproduct Lyase				
	SplA	63	65	73
	SplB	90	86	88
UVDE-dependent excision repair				
	YwjD	69	–	–
Y-family polymerase				
	PolY1 (YqjH)	70	71	75
	PolY2 (YqjW)	64	–	–
Alkyltransferases				
	Ogt	55	56	59
	YhaZ	54	–	–
Other				
	DinG	53	52	59
	End1 (YurI)	60	60	61
	KapD	65	66	77
	MutS2(YshD)	77	75	83
	YpcP	70	69	76
	YvcI	78	81	77
	YwqA	76	75	77

**Table 3:** Sequence conservation of H_2_O_2_ resistance proteins among *Bacillus* species

Function	Protein	% identity Bp vs. Bs	% identity Bp vs. Bl	% identity Bs vs. Bl
Catalase				
	KatX1 (YxlI)^1^	82	74	74
	KatX2^1^	47	48	74
	YjqC	79	64	62
	Mn-catalase^2^	25	24	78
Redox Proteins				
	BsaA	69	68	69
	Tpx	85	84	86
	TrxA	92	93	97
	TrxB	90	88	91
	YcgT	51	46	39
	YgaF	82	81	85
	YkuU	96	97	98
Organic hydroperoxide				
	OhrA	73	61	72
	OhrB	72	78	76
	OhrR	70	69	65
Transcription factors				
	PerR	93	92	91
	SigM (YhdM)	91	91	96
	SigB (RpoF)	87	84	86
	Spx (YjbD)	92	93	93
Other				
	DpsA (YktB)	82	82	78
Superoxide resistance				
	BcrC (YwoA)	50	47	60
	SodA	87	87	89
	SodF	56	60	66
	YojM	42	47	57
	YqjL	42	56	57

1 blastp vs. KatX.

2 blastp vs. YdbD.

Previous analyses of the resistance properties of *Bacillus* spores centered on small acid-soluble spore proteins (SASP) and the spore photoproduct lyase DNA repair system [26], [27]. SASP are spore core proteins that play a crucial role in resistance to UV radiation, heat, desiccation, and oxidative damage by binding DNA and altering its reactivity [5]. When exposed to UV radiation, SASP-bound DNA more readily forms the spore photoproduct (SP), 5-thyminyl-5,6-dihydrothymine, rather than cyclobutane dimers or (6-4)-photoproducts, which are formed in the absence of SASP. Unlike these other DNA lesions, SP is easily repaired by the spore photoproduct lyase (SP lyase), which is encoded by *splB* gene and is negatively regulated by the *splA* gene product [6]. *B. pumilus* has an intact *splAB* operon. The translated SplB (BPUM_1283) sequence is highly conserved in *B. pumilus*, but SplA (BPUM_1282) shows much more sequence diversity among *B. pumilus*, *B. subtilis*, and *B. licheniformis* (Table 2), indicating possible differences in SP lyase genetic regulation.

*Bacillus subtilis* produces 18 SASPs, whose sequences are short (40–100 amino acids) and highly conserved. The α/β-type SspA and SspB predominate, and there are also minor α/β-type SASPs, a γ–type, and novel SASPs [28]. Fifteen SASP genes were annotated in *B. pumilus* (Table 1); homologs of SspC, SspG, and SspH were not found. SspC is a minor α/β-type SASP that contributes to UV radiation resistance [29], hence its absence from *B. pumilus* is paradoxical. SspH and SspG are novel type SASPs, that have no effect on *B. subtilis* UV radiation resistance [28], [30]. The *B. pumilus* and *B. licheniformis* homologs of the γ–type SASP appear to be amino-terminal truncations of the 84 amino acid SspE of *B. subtilis*. The significance of such a truncation is unclear, as the only known function of SspE is as an amino acid source for germinating spores [31]. Although these differences in gene content and sequence conservation may contribute to the enhanced UV and oxidation resistance of *B. pumilus*, other important factors are likely to be found among DNA repair and oxidative stress response genes

### DNA Repair Mechanisms–Single Strand Repair pathways

#### Base Excision Repair (BER)

Oxidative damage to DNA is repaired by BER, which is performed by DNA glycosylases and AP (apurinic/apyrimidinic) endonucleases [32], [33]. DNA glycosylases remove damaged bases from the DNA backbone to create an AP site. AP endonucleases bind to this site and cleave the DNA 5′ to the abasic site, forming a free 3′-hydroxyl which is repaired by DNA polymerases. Monofunctional DNA glycosylases only have glycosylase activity, whereas bifunctional DNA glycosylases have both glycosylase and lyase activities as well as the ability to cleave the phosphodiester backbone 3′ to the AP site. *B. pumilus* encodes both monofunctional [AlkA (BPUM_0752), Ung (BPUM_03444)] and bifunctional [MutM (BPUM_2550), Nth (BPUM_1966)] DNA glycosylases in addition to the AP endonuclease IV, Nfo (BPUM_2246). Nth and Nfo are highly conserved among *B. pumilus*, *B. subtilis*, and *B. licheniformis*, but AlkA, Ung, and MutM have greater sequence divergence (Table 2).

*B. pumilus* lacks a homolog of the AP endonuclease ExoA and the DNA glycosylase YxlJ, both of which are present in *B. subtilis* and *B. licheniformis.* The lack of ExoA is not surprising, as *B. subtilis exoA* mutants do not exhibit enhanced sensitivity to H_2_O_2_ [34]. YxlJ functions in the repair of DNA alkylation damage and removal of deaminated purines and cyclic etheno adducts [35], and it is transcribed during spore germination and outgrowth [24]; its absence suggests that another protein compensates for its loss.

#### Nucleotide Excision Repair (NER)

While BER recognizes and repairs individual bases by specific DNA glycosylases, NER identifies multi-base distortions in the double helix and removes bulky single-stranded lesions, which are repaired by DNA polymerase I [36]. The *E. coli* NER pathway consists of UvrA and UvrB, which recognize DNA lesions, the UvrC nuclease, and the UvrD helicase. The NER machinery can be recruited to DNA damage by the Mfd protein in a process called transcription-coupled NER. In *B. subtilis*, NER is associated with UV radiation resistance in vegetative cells [26], and *uvrA* and *uvrB* are transcribed in germinating/outgrowing spores [24]. *B. subtilis* lacks UvrD, but likely uses PcrA to perform the UvrD helicase function [32], [37]. *B. pumilus* encodes homologs of UvrABC (BPUM_3147, 3148, & 2506), PcrA (BPUM_0625), and Mfd (BPUM_0039), the amino acid sequences of which are conserved with respect to *B. subtilis* and *B. licheniformis* (Table 2).

#### Mismatch Repair (MMR)

MMR recognizes and repairs mismatched bases in newly synthesized DNA daughter strands, and although not associated with DNA repair related to UV radiation or oxidative damage, it is important in maintaining genomic integrity [38]. In *E. coli*, MMR involves MutS and MutL, which recognize mismatches, and endonuclease MutH. *Bacillus* species lack MutH and must use another, unidentified mechanism [39]. *B. pumilus* MutS (BPUM_1608) and MutL (BPUM_1609) homologs are moderately well-conserved compared to those of *B. subtilis* and *B. licheniformis* (Table 2). Homologs of XseA (BPUM_2162) and XseB (BPUM_2161), subunits of a MMR exonuclease, were also annotated in *B. pumilus*.

### DNA Repair Mechanisms–Double Strand Repair pathways

#### Non-Homologous End-Joining (NHEJ)

The NHEJ pathway repairs double-strand DNA (DSB) breaks by directly joining DNA ends without requiring a homologous template to guide the repair [40]. Prokaryotic homologs of the eukaryotic DNA-end-binding protein, Ku, and DNA ligase IV were recently identified in several bacteria [41]. In *B. subtilis*, the NHEJ proteins are encoded on the *ykoUVW* operon, and *ykoU* and *ykoV* are transcribed both in vegetative cells and germinating/outgrowing spores [42]. *B. subtilis* YkoV (Ku) specifically recruits YkoU (DNA ligase IV) to DNA ends to stimulate DNA ligation, and loss of these proteins leads to hypersensitivity to UV radiation in *B. subtilis* [43]. YkoW is hypothesized to interact with dsDNA ends.

There is significant amino acid sequence variation in NHEJ proteins among *B. pumilus*, *B. subtilis*, and *B. licheniformis* (Table 2). The YkoV and YkoU sequences of *B. subtilis* and *B. licheniformis* are more closely related to each other than to their *B. pumilus* homologs (BPUM_1667 & BPUM_1666). Additionally, *B. pumilus* YkoW (BPUM_1234), at 807 amino acids in length, is much longer than *B. subtilis* YkoW (749 amino acids) and *B. licheniformis* (549 amino acids). Beyond these differences in amino acid sequences, which may affect protein function, the regulation of NHEJ genes appears to be different in *B. pumilus*. The *ykoUVW* operon structure of *B. subtilis* and *B. licheniformis* is not conserved in *B. pumilus*. In *B. pumilus, ykoU* and *ykoV* are adjacent and divergently transcribed, while *ykoW* is located on a separate locus.

#### Recombinational Repair

Homologous recombination (HR) is a ubiquitous process that is crucial for DNA repair and maintenance. It is a multi-step pathway involving several proteins that facilitate the invasion of dsDNA by a ssDNA substrate. As DNA is unwound by a helicase, the migrating strand replaces damaged DNA and the intermediate structure is resolved by an endonuclease [32]. HR in *Bacillus* species can be initiated by the AddAB pathway, which is analogous to *E. coli* RecBCD [44], or the RecFOR pathway [32]. Strand invasion and exchange is catalyzed by RecA, and *recA* mutations increase sensitivity to UV radiation [26]. Branch migration is performed by the RuvAB proteins, and resolution is performed by the RecU (RuvC in *E. coli*), and RecG proteins [32]. *B. pumilus* encodes homologs of all HR proteins common to *B. subtilis* and *B. licheniformis* (Tables 1 & 2).

Control of HR is closely related to the SOS repair system. In *B. subtilis*, the SOS regulon is similar to that in *E. coli* but it is also induced in competent cells in the absence of any DNA-damaging treatment [45]. RecA and the SOS transcriptional repressor LexA are the two main proteins involved in this coordinated cellular response to UV-light and DNA-damaging agents [46]. RecA is activated by ssDNA and promotes LexA self-cleavage, causing it to lose affinity to DNA and allowing expression of the SOS-response genes. The *B. pumilus* LexA (BPUM_1686) sequence is 87% similar to the *B. subtilis* and *B. licheniformis* LexA homologs, and their DNA-binding motifs are identical [47], suggesting that their activities are similar in these three species. Several SOS proteins have been identified in *E. coli* and *B. subtilis*, but the identification of *B. pumilus* SOS proteins will require experimental verification of regulation by RecA and LexA [46]. HR is also under the influence of RecX, a repressor of *recA* [48]. *B. pumilus* RecX (BPUM_0795) has moderate sequence conservation with its *B. subtilis* and *B. licheniformis* homologs.

### Other DNA repair systems

#### UVDE-dependent excision repair

YwjD is a *B. subtilis* homolog of UVDE, a eukaryotic protein that repairs UV radiation-induced cyclobutane pyrimidine dimers and 6-4 photoproducts [49]. *B. pumilus* encodes a YwjD (BPUM_3376) homolog that shares only moderate sequence identity with *B. subtilis* YwjD, which is produced in vegetative cells, and there is no *B. licheniformis* homolog. Because the sequence conservation is poor, it is possible that *B. pumilus* YwjD functions in a way that enhances its DNA repair activity.

#### Y-family polymerases

The Y family polymerases are error-prone, translesional DNA polymerases that are processive through DNA lesions that block the replicative polymerase [50]. Two Y-family polymerases were annotated in *B. pumilus* and named for their *B. subtilus* homologs, PolY1 (YqjH; BPUM_2125) and PolY2 (YqjW; BPUM_2102). In *B. subtilis* PolY2 is an SOS inducible polymerase that functions in UV damage repair and is necessary for UV-induced mutagenesis [50]. PolY2 is missing in *B. licheniformis,* which may contribute to its relative UV sensitivity. The fact that PolY2 is present in both *B. pumilus* and *B. subtilis* means that it alone cannot account for the UV resistance of *B. pumilus*. However, sequence variation (Table 2) and differences in expression may influence its activity. In contrast, PolY1 is a DinB subfamily polymerase that is constitutively expressed and functions in untargeted mutagenesis rather than UV-induced mutagenesis [50]. PolY1 is common to *B. pumilus*, *B. subtilis*, and *B. licheniformis*, and, therefore, unlikely to be responsible for UV resistance. Two other *B. subtilis* Y-family polymerases, UvrX and YozK-YobH are encoded on integrated prophages that are not present in *B. pumilus* [50].

#### Alkyltransferases

Alkylating chemicals can mutate DNA bases or the phosphodiester backbone by adding an alkyl group to the nitrogen or oxygen atoms. Ogt is a methyltransferase that removes the alkyl group from O^6^-alkyl guanine or, preferentially, O^4^-alkyl thymine [51]. Ogt also exhibits suicide inactivation by transferring an alkyl group to a cysteine residue in its own structure. Ogt (BPUM_1248) is found in *B*. *pumilus, B. subtilis* and *B. licheniformis*, although the protein sequence is not well-conserved (Table 2).

The *B. pumilus* genome encodes a second alkyltransferase, Ada (BPUM_1200), which, like Ogt, removes alkyl moities from DNA by suicide inactivation [32]. Ada also initiates the adaptive response, which activates several DNA repair enzymes [52]. There are notable differences between *B. pumilus* Ada and the homologs of *B. subtilis* and *B. licheniformis* that may be significant in DNA repair. *B. pumilus* Ada, like *E. coli* Ada, incorporates a regulatory domain fused to the alkyl glycosylase domain. However, in both *B. subtilis* and *B. licheniformis*, these domains are split into two proteins [53], AdaA and AdaB, neither of which align with greater than 50% identity to *B. pumilus* Ada. The fusion of the two proteins in *B. pumilus* raises the possibility that the function and transcriptional regulation of this alkyltransferase may be different in *B. pumilus* compared to *B. subtilis* and *B. licheniformis*.

#### Other proteins

The ATP-dependent DNA helicase DinG can unwind RNA or DNA, and it is a bacterial homolog of a human DNA repair helicase [54], [55]. Homologs of DinG (BPUM_1971) are present in *B. pumilus, B. subtilis* and *B. licheniformis,* although their sequences are not well conserved (Table 2).

The Nudix hydrolase superfamily MutT protein hydrolyzes 8-oxo-dGTP (a reactive oxygen species) and prevents its incorporation into DNA [56]. *B. subtilis* has three MutT superfamily genes, *mutT,* which is transcribed in germinating/outgrowing spores [24], *yjhB*, and *yvcI*. The *B. pumilus* genome has one *yvcI* gene (BPUM_3116), but no *mutT*; or *yjhB* homologs.

YshD is a MutS2 family protein that maintains genome integrity by inhibiting intergenomic recombination. The YshD (BPUM_2516) sequence is conserved among *B. pumilus*, *B. subtilis,* and *B. licheniformis* (Table 2), but it is unclear if it has an effect on UV or H_2_O_2_ resistance.

### DNA repair proteins unique to B. pumilus

The *B. pumilus* genome encodes PhrB (BPUM_1378), a DNA photolyase enzyme that repairs cyclobutane-pyrimidine dimers [57]. Although no homolog exists in *B. subtilis* and *B. licheniformis*, there are homologs in other *Bacillus* species such as *B. firmus*, *B. cereus, B. anthracis*, and *B. thuringiensis*. Nevertheless, none of these species exhibit UV radiation resistance comparable to *B. pumilus*. The *B. subtilis* photolyase amino acid sequence is diverse with respect to other photolyases. It shares 32% amino acid identity with *E. coli* PhrB and only 46% sequence identity with its closest homolog from *B. firmus*. It is logical that the presence of a photolyase gives *B. pumilus* UV resistance capabilities that *B. subtilis* and *B. licheniformis* lack. However, because less UV-resistant *Bacillus* species also have photolyase enzymes, the relation of photolyase to enhanced UV resistance is not clear. Although the sequence divergence in the *B. pumilus* photolyase may indicate altered function, *B. pumilus* may rely on a combination of other factors for its UV resistance properties.

Genes encoding two DNA repair/modification proteins not found in *B. subtilis* and *B. licheniformis* were also annotated in *B. pumilus*. One sequence (BPUM_0608) is similar to a Superfamily II (SF-2) helicase based upon the presence of a DExD Walker B motif in conserved motif II [58]. SF-2 helicases are known to function in NER and recombinational repair in yeast [59]. Although it cannot be predicted that this helicase functions in DNA repair, if it does have such a function it would be a feature that *B. subtilis* and *B. licheniformis* lack, possibly contributing to the enhanced UV radiation resistance. *B. pumilus* also encodes a C-5 cytosine-specific DNA methyltransferase (BPUM_0656) that has no homolog in either *B. subtilis* or *B. licheniformis*. Though unlikely to be directly implicated in DNA repair, it is possible that a unique DNA-modifying protein may contribute to genomic stability in *B. pumilus*. Additionally, the *B. pumilus* genome has 517 coding sequences that are not common to *B. subtilis* and *B. licheniformis*, including 218 hypothetical proteins that have no sequence similarity to any known sequence in the nr database. It is possible that one more of these coding sequences of unknown function may contribute to UV radiation resistance.

### H_2_O_2_ resistance

*Bacillus* species use a variety of proteins to resist the toxic effects of H_2_O_2_, including catalases, and various reducing proteins such as alkyl hydroperoxide reductase and peroxiredoxins [60]. Analysis of the *B. pumilus* genome reveals many striking differences compared to similar proteins in *B. subtilis* and *B. licheniformis*.

#### Catalase

Catalases convert H_2_O_2_ into water and oxygen in a highly efficient reaction that requires neither ATP nor an exogenous reducing agent [8]. *B. subtilis* and *B. licheniformis* produce two vegetative catalases, KatA and KatB (KatE), and one germination catalase, KatX, which is present in spores and protects germinating cells from H_2_O_2_ [61]. All three catalases are transcribed in germinating/outgrowing spores [24]. *B. pumilus* has no homolog to either vegetative catalase, however, it has two KatX homologs. The sequence conservation of KatX1 (BPUM_3712) is moderate, but KatX2 (BPUM_0892) is more diverse, sharing less than 50% identity with *B. subtilis* and *B. licheniformis* KatX. A second germination-specific catalase with substantial sequence diversity is a candidate protein that may explain the enhanced peroxide resistance of *B. pumilus* spores.

YjqC and YdbD are two additional proteins with catalase domains that are found in *B. subtilis* and *B. licheniformis*, although little is known of their functions. *B. pumilus* YjqC (BPUM_2346) shares moderate sequence identity with its *Bacillus* homologs (Table 3), but there is no YdbD homolog in *B. pumilus*. However, a *B. pumilus* sequence containing a manganese catalase domain does exist (YdbD uses Mn^2+^ as a cofactor). It is possible that this catalase (BPUM_1305), which differs greatly from YdbD, may have properties that contribute to the H_2_O_2_ resistance of *B. pumilus*.

The spore coat protein CotJC contains a predicted catalase domain in its amino acid sequence. Although CotJC is present in *B. subtilis* and *B. licheniformis*, no homolog was identified in *B. pumilus*, suggesting that it is not necessary for elevated peroxide resistance.

#### Peroxiredoxins

Bacteria use peroxiredoxins to reduce H_2_O_2_ to water [62]. Four peroxiredoxins were annotated in *B. pumilus*. Three peroxiredoxin protein sequences, YkuU (BPUM_1319), YgaF (BPUM_0826), and Tpx (BPUM_2581), are highly conserved with respect to their *B. subtilis* and *B. licheniformis* homologs (Table 3). The fourth peroxiredoxin (BPUM_3690) annotated in *B. pumilus* has no obvious homolog in *B. subtilis* or *B. licheniformis*. Instead, these two species produce an alkyl hydroperoxide reductase that is induced upon H_2_O_2_ stress. The enzyme is a heterodimer of AhpC and AhpF, and it uses NADH or NADPH as a reducing agent. In *B. subtilis* and *B. licheniformis* the subunits are encoded on the *ahpCF* operon, and their translated sequences are highly conserved (>90% identity for AhpC and AhpF). The *B. pumilus* genome does not contain a homologous operon. However, it does it does have a gene encoding an NADH dehydrogenase (BPUM_2106), which, if coupled with the peroxiredoxin (BPUM_3690), could hypothetically function as an alkyl hydroperoxide reductase. Given the lack of sequence and gene order conservation, the function may be distinct from *B. subtilis* and *B. licheniformis* AphCF, possibly explaining the abnormal H_2_O_2_ resistance of *B. pumilus*.

#### Peroxidases

Peroxidases also reduce H_2_O_2_ to water using NADH or NADPH as a cofactor [8]. A glutathione peroxidase, BsaA (BPUM_1925), was annotated in *B. pumilus*. BsaA uses glutathione as a reducing agent to reduce lipid hydroxyperoxides formed by peroxide stress, and *bsaA* is transcribed during spore germination/outgrowth [24]. There is substantial sequence diversity among the BsaA homologs of *B. pumilus*, *B. subtilis*, and *B. licheniformis* (Table 3), raising the possibility that differences in *B. pumilus* BsaA may contribute to H_2_O_2_ resistance.

#### Other reducing agents

Thioredoxins and glutaredoxins are instrumental to peroxide stress resistance. They reduce peroxiredoxins and peroxidases, facilitating their functions, and act as hydroxyl radical scavengers [63]. They also maintain oxidation states of cytoplasmic proteins, preventing illegitimate disulfide bond formation [64]. Several redox proteins were annotated in *B. pumilus*, but only those known to be related to peroxide stress and those unique compared to *B. subtilis* and *B. licheniformis* are mentioned in this work.

TrxA is the product of the thioredoxin A gene, which is essential in *B. subtilis*. The reducing potential of TrxA is recycled by the thioredoxin reductase, TrxB. *B. pumilus* TrxA (BPUM_2507) and TrxB (BPUM_3117) share approximately 90% identity with their *B. subtilis* homologs. A second TrxB-like thioredoxin reductase (BPUM_0664) was annotated in *B. pumilus* (Table 4). This protein has no *B. subtilis* homolog and a poor alignment to a *B. licheniformis* reductase, so it may provide peroxide stress resistance capabilities not available to these species. YcgT (BPUM_0777), another thioredoxin-disulfide reductase, is present in *B. pumilus*, but its sequence is not well-conserved with *B. subtilis* and *B. licheniformis*, raising the possibility that differences in YcgT activity may be important to *B. pumilus* oxidative stress resistance.

**Table 4:** Unique DNA repair and H_2_O_2_ resistance proteins of *B. pumilus*.

BPU locus tag^2^	Definition
DNA repair	
BPUM_1378	photolyase PhrB
BPUM_1200	DNA repair methyltransfrease Ada
BPUM_0608	helicase
BPUM_0656	DNA (cytosine-5-)-methyltransferase
H_2_O_2_ resistance	
BPUM_0664	TrxB-like thioredoxin-disulfide reductase^1^
BPUM_0931	lysine/ornithine N-monooxygenase
BPUM_1716	NADH-dependent flavin oxidoreductase^2^
BPUM_2106	NADH dehydrogenase
BPUM_3690	peroxiredoxin
BPUM_1153	possible FAD dependent oxidoreductase
BPUM_1731	flavin reductase
BPUM_0802	possible monooxygenase
BPUM_0482	probable dioxygenase
BPUM_3130	thioredoxin

1 homolog in *B. licheniformis*, but not *B. subtilis.*

2 only 42% identity with *B. licheniformis* and *B. subtilis* YqjM; *B. pumilus* YqjM found at BPUM_2112.

#### The *ohr* operon

In *B. subtilis* resistance to organic peroxides is encoded by the *ohr* locus, which produces the peroxide resistance proteins OhrA and OhrB, and OhrR, a transcriptional regulator of *ohrA* [65]. The *B. pumilus* homologs (BPUM_1211-1213) of these proteins share moderate homology with their *B. subtilis* and *B. licheniformis* homologs (Table 3), so they may have altered function due to sequence diversity.

#### Regulation of the oxidative stress response

Several transcriptional regulators of the *B. subtilis* oxidative stress response are known, including PerR, Spx, and sigma factors SigM and SigB. All four of these proteins are conserved in *B. pumilus* (Table 3). Dps proteins are DNA- binding proteins that protect bacteria from oxidative stress by sequestering iron and oxidants and storing them as benign ferric oxide minerals [66]. Two Dps proteins, DpsA (YktB) and MrgA are known in *B. subtilis* and *B. licheniformis*. *B. pumilus* does encode a DpsA homolog (Table 3; BPUM_2703), however, it has no MrgA homolog. Although MrgA is important for peroxide resistance proteins in vegetative cells, it has no effect on peroxide resistance in spores [67].

#### Other oxidative stress proteins

Oxidative stress also occurs in the form of superoxide, O_2_^−^. Although the O_2_^−^ and H_2_O_2_ stress responses are distinct, the conditions are related, via the chemical conversion of O_2_^−^ to H_2_O_2_ by superoxide dismutases. *B. pumilus* has three superoxide dismutases: SodA (BPUM_2230), which uses a manganese cofactor, SodF (BPUM_1859), which uses an iron cofactor, and YojM (BPUM_1865), which uses copper or zinc as a cofactor [8]. *B. pumilus* SodA is highly conserved with respect to the homologs of *B. subtilis* and *B. licheniformis*, but there is much greater sequence diversity in SodF and YojM (Table 3). If these changes in sequence have any effect on protein function, it is difficult to speculate what benefit there would be for H_2_O_2_ resistance, as any decrease in superoxide reductase-mediated H_2_O_2_ production would mean less efficient removal of O_2_^−^. The hydrolase YqjL (BPUM_2113) and the efflux protein BcrC (BPUM_3294) both contribute to O_2_^−^ resistance by unknown mechanisms [68]. The *B. pumilus* homologs of these proteins are not well-conserved with respect to their *B. subtilis* and *B. licheniformis* homologs (Table 3). It is possible that *B. pumilus* YjqL and/or BcrC may be more adept at O_2_^−^ detoxification than their *B. subtilis* and *B. licheniformis* homologs, and that their activities may alleviate the production of H_2_O_2_ by superoxide dismutases.

Two additional proteins related to oxidative stress resistance in *B. subtilis* were not annotated in the *B. pumilus* genome. YlaC is a *B. subtilis* extracytoplasmic sigma factor that is regulated by the anti-sigma factor YlaD, which contains an oxidative stress-sensing domain [69]. Transcription of *ylaCD* was also shown to be Spx-dependent, further linking it to the oxidative stress response. Nevertheless, the lack of YlaC and YlaD homologs in *B. pumilus* indicates that despite the function of these proteins, they are not essential to H_2_O_2_ resistance.

The database of annotated *B. pumilus* coding sequences was examined for oxygenases, oxidoreductases, and redoxins without homologs in *B. subtilis* or *B. licheniformis*. The predicted proteins and functions associated with these regions are listed in Table 4. Additionally, it is possible that proteins functioning in peroxide resistance are among the hypothetical proteins and other undefined ORFs that have no *B. subtilis* or *B. licheniformis* homolog.

### Conclusion

Given the phenotypic differences between *B. pumilus* and *B. subtilis* and *B. licheniformis* in terms of UV radiation resistance and H_2_O_2_ resistance, it was expected that a comparison of the genomes of these species would point to unique *B. pumilus* genes related to these functions. Most genes related to DNA repair and H_2_O_2_ resistance are conserved among these species. Paradoxically, *B. pumilus* lacks several DNA repair and oxidative stress response genes found in *B. subtilis* and *B. licheniformis*. Nevetheless, this analysis has identified several *B. pumilus* genes worthy of further study because of their absence in related organisms, differences in amino acid sequence, or predicted differences in genetic regulation.

## Supporting Information

Table S1 Locus tag numbers of genes in Table 1.
(0.09 MB DOC)
