# Supplementary material for: Paradoxical DNA Repair and Peroxide Resistance Gene Conservation in Bacillus pumilus SAFR-032
Source: PLoS One. 2007 Sep 26;2(9):e928. doi: 10.1371/journal.pone.0000928 (PMC1976550; doi:10.1371/journal.pone.0000928)
Supplement: Table S1 — Locus tag numbers of genes in Table 1. (0.09 MB DOC) [file pone.0000928.s001.doc]

**Table S1: Locus tag numbers of genes in Table 1**.

| **Gene name** | **Locus tag** | **Gene name** | **Locus tag** |
| --- | --- | --- | --- |
| *ada* | BPUM_1200 | *sodA* | BPUM_2230 |
| *addA* | BPUM_0994 | *sodF* | BPUM_1859 |
| *addB* | BPUM_0993 | *splA* | BPUM_1282 |
| *alkA (yfiP)* | BPUM_0752 | *splB* | BPUM_1283 |
| *bcrC (ywoA)* | BPUM_3294 | *spx (yjbD)* | BPUM_1077 |
| *bsaA* | BPUM_1925 | *ssb* | BPUM_3719 |
| *dinG* | BPUM_1971 | *sspA (sspBA)* | BPUM_2601 |
| *disA (yacK)* | BPUM_0073 | *sspB* | BPUM_0921 |
| *dnaE* | BPUM_2566 | *sspD* | BPUM_1239 |
| *dpsA (yktB)* | BPUM_2703 | *sspE* | BPUM_0813 |
| *end1 (yurI)* | BPUM_2907 | *sspF* | BPUM_0029 |
| *gyrA* | BPUM_0007 | *sspI* | BPUM_2524 |
| *gyrB* | BPUM_0006 | *sspJ* | BPUM_2997 |
| *hbs* | BPUM_2010 | *sspK* | BPUM_0798 |
| *kapD* | BPUM_2815 | *sspL* | BPUM_1936 |
| *katX1 (yxlI)* | BPUM_3712 | *sspM* | BPUM_1961 |
| *katX2* | BPUM_0892 | *sspN* | BPUM_1702 |
| *lexA (dinR)* | BPUM_1686 | *sspO (cotK)* | BPUM_1698 |
| *mfd* | BPUM_0039 | *sspP (cotL)* | BPUM_1697 |
| *msrA* | BPUM_1901 | *tlp* | BPUM_1703 |
| *msrB (yppQ)* | BPUM_1900 | *topA* | BPUM_1510 |
| *mutL* | BPUM_1609 | *tpx* | BPUM_2581 |
| *mutM* | BPUM_2550 | *trxA* | BPUM_2507 |
| *mutS1* | BPUM_1608 | *trxB* | BPUM_3117 |
| *mutS2 (yshD)* | BPUM_2516 | *ung* | BPUM_3444 |
| *mutY (yfhQ)* | BPUM_0810 | *uvrA* | BPUM_3147 |
| *nfo (yqfS)* | BPUM_2246 | *uvrB* | BPUM_3148 |
| *nth* | BPUM_1966 | *uvrC* | BPUM_2506 |
| *ogt* | BPUM_1248 | *xseA (yqiB)* | BPUM_2162 |
| *ohrA* | BPUM_1211 | *xseB (yjiC)* | BPUM_2161 |
| *ohrB* | BPUM_1213 | *ycgT* | BPUM_0777 |
| *ohrR* | BPUM_1212 | *ydiP* | BPUM_0561 |
| *pcrA* | BPUM_0625 | *ygaF* | BPUM_0826 |
| *pcrB* | BPUM_0624 | *yhaZ* | BPUM_3600 |
| *perR* | BPUM_0827 | *yjcD* | BPUM_1114 |
| *phrB* | BPUM_1378 | *yjqC* | BPUM_2346 |
| *polA* | BPUM_2551 | *ykoU (lig)* | BPUM_1666 |
| *polY1 (yqjH)* | BPUM_2125 | *ykoV (ku)* | BPUM_1667 |
| *polY2 (yqjW)* | BPUM_2102 | *ykoW* | BPUM_1234 |
| *priA (srgA)* | BPUM_1470 | *ykuU* | BPUM_1319 |
| *radC (ysxA)* | BPUM_2444 | *ylbH* | BPUM_1394 |
| *recA* | BPUM_1598 | *yneB* | BPUM_1688 |
| *recD (yrrC)* | BPUM_2389 | *yocI* | BPUM_1852 |
| *recF* | BPUM_0004 | *yojM* | BPUM_1865 |
| *recG (ylpB)* | BPUM_1486 | *ypcP* | BPUM_1937 |
| *recJ (yrvE)* | BPUM_2403 | *yprA* | BPUM_1954 |
| *recN* | BPUM_2156 | *ypvA* | BPUM_1946 |
| *recO* | BPUM_2260 | *yqfN* | BPUM_2251 |
| *recQ (recS, ypbC)* | BPUM_2035 | *yqhH* | BPUM_2190 |
| *recR* | BPUM_0518 | *yqjL* | BPUM_2113 |
| *recU* | BPUM_1963 | *yqjM* | BPUM_2112 |
| *recX (yfhG)* | BPUM_0795 | *yrrK* | BPUM_2378 |
| *ruvA* | BPUM_2416 | *yrrT* | BPUM_2363 |
| *ruvB* | BPUM_2415 | *yrvN* | BPUM_2394 |
| *sbcC (yirY)* | BPUM_0996 | *ytkD* | BPUM_2697 |
| *sbcD* | BPUM_0995 | *yvcI* | BPUM_3116 |
| *scpA* | BPUM_2055 | *ywbD* | BPUM_3488 |
| *scpB (ypuH)* | BPUM_2054 | *ywjD (uvsE)* | BPUM_3376 |
| *sigB (rpoF)* | BPUM_0446 | *ywqA* | BPUM_3279 |
| *sms (radA)* | BPUM_0072 |  |  |
